# Genomic insights into Yak (*Bos grunniens*) adaptations for nutrient assimilation in high-altitudes

**DOI:** 10.1038/s41598-024-55712-3

**Published:** 2024-03-07

**Authors:** Hafiz Ishfaq Ahmad, Sammina Mahmood, Mubashar Hassan, Muhammad Sajid, Irfan Ahmed, Borhan Shokrollahi, Abid Hussain Shahzad, Shaista Abbas, Sanan Raza, Komal Khan, Sayyed Aun Muhammad, Dalia Fouad, Farid S. Ataya, Zhengtian Li

**Affiliations:** 1 Department of Animal Breeding and Genetics, Faculty of Veterinary and Animal Sciences, The Islamia University of Bahawalpur, Bahawalpur, Pakistan; 2 Department of Botany, Division of Science and Technology, University of Education, Lahore, Pakistan; 3 Department of Clinical Sciences, College of Veterinary and Animal Sciences (Sub campus UVAS, Lahore), Jhang, 35200 Pakistan; 4 Department of Pathobiology, College of Veterinary and Animal Sciences (Sub campus UVAS, Lahore), Jhang, 35200 Pakistan; 5 Department of Animal Nutrition, Faculty of Veterinary and Animal Sciences, The Islamia University of Bahawalpur, Bahawalpur, Pakistan; 6 Hanwoo Research Institute, National Institute of Animal Science, Pyeongchang, 25340 Korea; 7 Department of Physiology and Biochemistry, College of Veterinary and Animal Sciences, Jhang, 35200 Pakistan; 8 Department of Basic Sciences, Anatomy Section, College of Veterinary and Animal Sciences, Jhang, 35200 Pakistan; 9 Department of Zoology, College of Science, King Saud University, PO Box 22452, Riyadh, 11495 Saudi Arabia; 10 Department of Biochemistry, College of Science, King Saud University, PO Box 2455, 11495 Riyadh, Saudi Arabia; 11 Qujing Normal University, College of Biological Resource and Food Engineering, 655011 Yunnan, China

**Keywords:** High-altitude adaptation, Yak genomics, Nutrient assimilation, Positive selection, Evolutionary mechanisms, Computational biology and bioinformatics, Evolution, Genetics, Physiology

## Abstract

High-altitude environments present formidable challenges for survival and reproduction, with organisms facing limited oxygen availability and scarce nutrient resources. The yak (*Bos grunniens*), indigenous to the Tibetan Plateau, has notably adapted to these extreme conditions. This study delves into the genomic basis of the yak’s adaptation, focusing on the positive selection acting on genes involved in nutrient assimilation pathways. Employing techniques in comparative genomics and molecular evolutionary analyses, we selected genes in the yak that show signs of positive selection associated with nutrient metabolism, absorption, and transport. Our findings reveal specific genetic adaptations related to nutrient metabolism in harsh climatic conditions. Notably, genes involved in energy metabolism, oxygen transport, and thermoregulation exhibited signs of positive selection, suggesting their crucial role in the yak’s successful colonization of high-altitude regions. The study also sheds light on the yak's immune system adaptations, emphasizing genes involved in response to various stresses prevalent at elevated altitudes. Insights into the yak’s genomic makeup provide valuable information for understanding the broader implications of high-altitude adaptations in mammalian evolution. They may contribute to efforts in enhancing livestock resilience to environmental challenges.

## Introduction

Domestic yaks (*Bos grunniens*) are integral to the livelihoods of communities residing in the Tibetan Plateau's high-altitude regions and its surrounding mountainous areas^1^. Their comparison with low-altitude relatives, taurine cattle *(Bos taurus*), provides a unique opportunity to study adaptation to high-altitude challenges^2^. These challenges predominantly arise from limited oxygen availability, which the yak has overcome through distinct physiological adaptations developed over time^3,4^.

In regions like northern Pakistan, with potential impacts of climate change, such as reduced glacial meltwater feeding into rivers^5^, maintaining agricultural activities may become increasingly difficult. Here, preserving domestic yaks becomes a strategic approach for risk mitigation, as they are well adapted to high-altitude environments, ranging from 2000 to 5000 m above sea level, across the Hindu Kush, Karakoram, Himalayan, and Pamir mountains^6^. Animals acclimate to a specific stressor or a variety of stressors in order to adapt to their living conditions and external stress^7^. While adaptation can be essential for life, it frequently has a detrimental impact on livestock systems' profitability and production. The capacity to adapt is influenced by morphological and physiological changes that help animals better adapt for survival, as well as the adaptability of behavioral features^8^. For instance, types of sheep known as fat tails or fat rump comprise almost 25% of the global sheep population. These breeds are adapted to tough semi-arid desert conditions with variable food supply. When food is scarce, the animal’s fat tail or rump serves as storage, allowing it to live for extended periods^9^. In light of the constantly shifting climate, heat stress is a major concern these days. The effects of heat stress on ruminant animals are well established and include reduced growth, reproduction, and productivity, as well as an increase in health problems and mortality^10^. Sheep and goats, on the other hand, are less vulnerable to environments that are hot than other ruminant species^11^. The primary methods of small ruminants’ adaptation to heat-stressed habitats include behavioral, morphological, physiological, and genetic basis^12^.

In particular, the yak’s specialized oxygen metabolism enables its survival at altitudes above 4000 m, distinguishing it from other livestock. The Gilgit-Baltistan (GB) region in Pakistan hosts a significant population of both yak hybrids and pure yaks, contributing to the local Tibetan- and Balti-speaking Muslim communities’ sustenance^13^. These hybrid yaks, especially F1 hybrids, show adaptability to lower elevations, offering utility for agricultural tasks. Female hybrids are particularly valued for their higher milk production compared to pure yaks^14^.

Despite these advantages, when taurine cattle are introduced to yak habitats, they suffer from severe pulmonary hypertension, showcasing the yak’s unique adaptations. These adaptations include oversized lungs and hearts, exceptional foraging abilities, environmental adaptability, rapid energy metabolism, and the absence of hypoxic pulmonary vasoconstriction^15^. Additionally, the role of the gut microbiome in yak adaptation to high-altitude environments has recently gained attention, given its essential functions in immune system development, metabolic regulation, and vitamin biosynthesis^16,17^. However, our understanding of how domestication impacts the gut microbiome of herbivorous mammals is still in its infancy^18^. The frequency of genetic variations in a population may fluctuate due to artificial or natural selection pressure. Selection may be balancing, negative, or positive^12^. A population's frequency of fitness-enhancing variations rises under positive selection, while unfavorable mutations are eliminated under negative selection to preserve the functional integrity of DNA^19^. When heterozygotes have a higher fitness level, balancing selection keeps more than one allele of the gene^20^. Through the hitch-hiker effect, the genes in the genomic region in linkage disequilibrium with the genes under selection would likewise experience an increase or decrease in frequency^21^, altering the anticipated patterns of molecular variation and providing a “selection signature”.

Comprehending the hypoxic aerobic metabolism of yaks can yield significant understandings regarding adaptive evolution. These discoveries, along with cutting-edge molecular methods and genetic studies, offer a foundation for examining the genetic processes that underlie climate change adaptation—a current research focus. While domestic yaks exhibit distinct genetic and environmental adaptations compared to their wild counterparts, the yaks (*Bos mutus*), there is a loss of genes related to sugar metabolism and a selection for tameness genes^22,23^. This study aims to delve deeper into the genetic basis of yak adaptation to high-altitude environments, focusing on the digestive enzyme genes isolated from Yak genomes and examining the selective pressures these genes face due to the yaks’ diverse diet.

## Results

### Genomic comparisons and adaptation to high altitude

A comparative analysis between cattle and yak genomes revealed a pronounced divergence, highlighting the yak’s unique adaptation to high-altitude living. Specifically, we identified an increased number of gene families in yaks that are predominantly associated with sensory perception and energy metabolism. This genomic characteristic suggests a potential evolutionary response to the challenges posed by hypoxic conditions at high altitudes. Furthermore, there is a noticeable enrichment in protein domains within the yak lineage that play critical roles in detecting hypoxic stress and monitoring extracellular conditions, illustrating a sophisticated biological adaptation to their environment.

### Functional categories and pathways related to nutrition and hypoxia

Our findings underscore a substantial enrichment in genes that have undergone positive selection and rapid evolution, falling predominantly within functional categories and pathways related to nutrition metabolism and hypoxic response. These genetic adaptations are crucial for yaks, as they enhance their ability to efficiently metabolize nutrients and thrive under low oxygen conditions, which are characteristic of high-altitude habitats. This insight not only advances our understanding of animal adaptation to extreme environments but also has potential implications for addressing hypoxia-related human disorders.

### Evolutionary history of nutritional pathway genes

In our pursuit to unravel the evolutionary history of nutritional pathway genes across mammals, we conducted comprehensive evolutionary analyses on two digestive enzyme genes present in 48 mammalian genomes, using the yak as our reference genome (Supplementary file). This analysis, summarized in Table 1, is crucial for understanding how these genes have evolved and adapted to diverse dietary needs, particularly in species inhabiting high-altitude regions. The selective pressure assessments indicated that these digestive enzyme genes had undergone adaptive evolution, which may be attributed to dietary diversification in these species.

**Table 1:** aBSREL discovered evidence of episodic diversifying selection on 10 out of 57 branches in the phylogeny.

Gene	Model	Log (L)	AIC-c	Parameters	Rate distribution	Rate plot
CAMK2B	Unconstrained	− 18,768.3	37,703	83	Tested ω, 0.005944 (79.310%) 0.4217	1,000,200,030,004,000
					(20.143%) 4002 (0.54791%)	
					Mean = 22.02, CoV = 13.42	
	Unconstrained				Synonymous rates	0.00.51.01.52.0
					0.08229 (17.572%) 1.097 (75.725%) 2.311 (6.7038%)	
					Mean = 1.000, CoV = 0.5199	
	Constrained	− 18,942.1	38,048.5	82	Tested ω, 0.008774 (83.708%)	0.00.20.40.60.81.0
					1.000 (3.2128%) 1.000 (13.079%)	
					Mean = 0.1703, CoV = 2.150	
	Constrained				Synonymous rates	0
					0.1694 (20.983%) 0.9237 (55.397%) 1.917 (23.620%)	
					Mean = 1.000, CoV = 0.5886	
GLUL	Constrained	− 3326.42	6737.65	42	Tested ω	0.00.20.40.60.81.0
					0.000 (72.018%) 0.05540 (24.530%) 1.000 (3.4528%)	
					Mean = 0.04812, CoV = 3.773	
	Constrained	− 3324.56	6735.96	43	Synonymous rates	1234
					0.1992 (24.062%) 0.9331 (67.475%) 3.810 (8.4633%)	
					Mean = 1.000, CoV = 0.9086	
	Unconstrained				Tested ω	12,345
					0.000 (77.293%) 0.09558 (21.695%) 4.649 (1.0123%)	
					Mean = 0.06780, CoV = 6.858	
	Unconstrained				Synonymous rates	0.00
					0.06837 (10.292%) 0.7386 (74.795%) 2.954 (14.914%)	
					Mean = 1.000, CoV = 0.8424	

### Positive selection in yak nutrition pathway genes

Our data highlight the pivotal role of positive selection in the adaptive evolution of nutrition pathway genes in yaks, contributing to their enhanced nutritional assimilation at high altitudes. Notably, genes such as the lactase gene have undergone positive selection, enabling yaks to continue lactase production into adulthood and efficiently digest lactose-rich dairy products. This adaptation is crucial for their survival, providing them with a reliable source of essential nutrients in an otherwise challenging environment. Furthermore, genes involved in the metabolism of fatty acids, carbohydrates, and amino acids have also shown signs of positive selection, further supporting the yak's remarkable ability to thrive at high altitudes.

### Structural analysis of protein kinases

**Figure 1:**
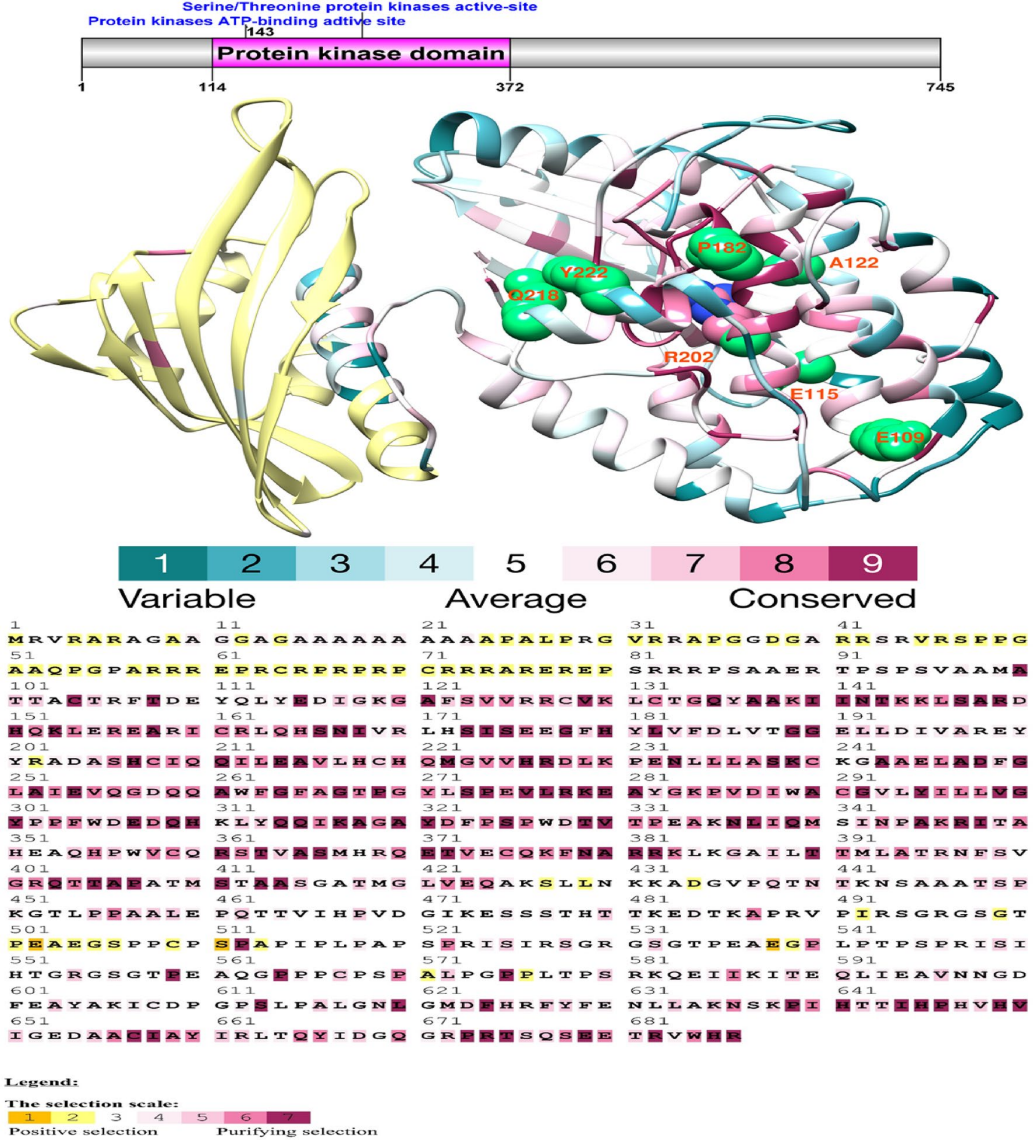
Domain architecture and selection analysis of the yak CAMK2B protein. This figure, created with the DOG 1.0 illustrator, showcases the domain structure of the CAMK2B protein and emphasizes the analysis of its conserved domains. Special attention is given to the protein kinase domain, where sites under positive selection have been identified. These sites are mapped onto the three-dimensional structure of the yak CAMK2B protein, revealing the adaptive evolution at the molecular level. The Selecton analysis is visualized through a color-coded scheme, where sequences are compared against aligned nucleotide coding sequences to infer selection pressures. Codons under positive selection are highlighted in yellow and brown, indicating adaptive changes. Codons under neutral selection are marked in grey and white, signifying evolutionary neutrality, and codons under purifying selection are in purple, denoting the removal of deleterious mutations. This color coding allows for an at-a-glance understanding of the selective forces acting on the CAMK2B protein.

**Figure 2:**
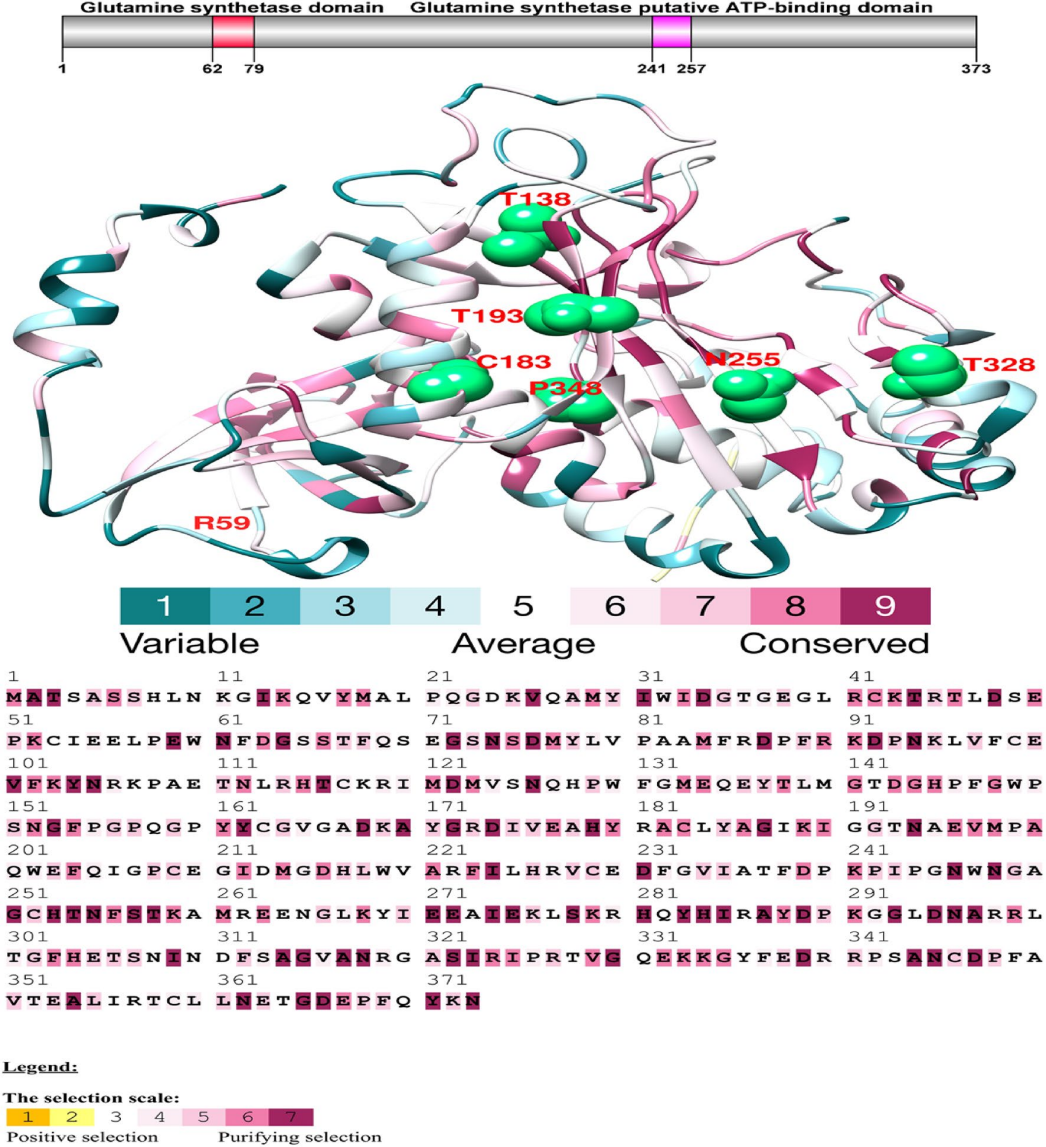
Domain structure analysis of the yak glul protein**.** Illustrated using the DOG 1.0 illustrator, this figure represents the molecular structure of the GLUL protein and its functional domains, with a focus on the analysis of conserved domains. Notably, sites of positive selection have been identified within the ATP binding domain. The three-dimensional structure of the yak GLUL protein has been annotated to indicate positively selected amino acid sites derived from Selecton analysis. The figure employs a color-coding scheme to represent the type of selection pressure on codons: yellow and brown for positive selection, suggesting adaptive evolutionary changes; grey and white for neutral selection, indicating evolutionary stasis; and purple for purifying selection, where detrimental variations are selectively eliminated. This color-coding facilitates a visual distinction between the different selection pressures across the protein's structure.

The structural analysis of protein kinases has revealed conserved regions critical for their function. As illustrated in Figs. 1 and 2, the catalytic domain of these kinases features several conserved areas. Two specific locations have been identified with signature patterns: a glycine-rich stretch near the N-terminal end crucial for ATP binding and a conserved aspartic acid residue in the middle region vital for catalytic activity. Understanding these conserved patterns provides insight into the molecular mechanisms driving the adaptive evolution of yaks, offering a basis for future studies aiming to unravel the complexities of life at high altitudes.

Furthermore, we investigated adaptive selection utilizing the MG94xREV baseline model, which estimates a single rate category for each branch. Subsequently, we employed the adaptive aBSREL model, known as the fully adaptive model, to determine the optimal number of rate categories for each branch. Notably, ABSREL detected evidence of episodic diversifying selection among ten of the phylogeny’s 57 branches. A formal testing of diversifying selection was conducted on all 57 branches. To assess the statistical significance of the results, we employed the Likelihood Ratio Test, considering a significance level of *p* < 0.05, while adjusting for the impact of repeated tests (Table 1). The detailed data table provides insights into the significance and the number of rate categories inferred for each branch.

### Adaptive evolution and diversifying selection

Yaks (*B. grunniens*) are domesticated animals well adapted to life at high altitudes in the Himalayan region. An essential aspect of their adaptation is their efficient assimilation of nutrients from their primarily low-quality forage diet. Our analyses indicate that yaks have undergone adaptive evolution in their nutrition pathway genes, enhancing nutrient assimilation at high altitudes.

Specifically, genes responsible for carbohydrate and lipid metabolism, as well as nutrient absorption and transport, have experienced positive selection in yaks compared to their counterparts at lower altitudes, such as cattle. This adaptive evolution has enabled yaks to thrive in high-altitude environments. For example, the CAMK2B and GLUL genes, encoding calcium/calmodulin-dependent protein kinase II beta and glutamine synthetase, a crucial enzyme in nitrogen metabolism, belong to the CaM kinase family. These genes have undergone positive selection in yaks (*B. grunniens*), highlighting their importance in yak adaptation to high altitudes.

Our research identifies several distinct amino acid changes in the CAMK2B and GLUL genes of yaks compared to those in cattle. These modifications could impact the structure and function of CAMK2B and GLUL proteins, affecting their activity and regulation. This suggests that the unique physiological and metabolic adaptations that enable yaks to thrive at high altitudes may be the result of the adaptive evolution of CAMK2B and GLUL genes.

We conducted phylogenetic tree analysis using BUSTED to detect instances of positive selection at various nodes. This analysis assessed the ratio of synonymous to non-synonymous changes at each site to determine whether those sites experienced positive selection. The tests considered three classes of non-synonymous rate variation across 59 median branches/partitions. Our investigation, based on the likelihood ratio test, identified indications of episodic diversifying selection in the dataset (Table 2).

**Table 2:** Detailed site-by-site results from the FEL analysis.

Gene	Partition	Codon	Alpha	Beta	Alpha = Beta	LRT	*p*-value	Branch length	dN/dS LB	dN/dS MLE	dN/dS UB	Class
CAMK2B	1	506	0	0.804	0.507	5.387	0.0203	3995.783	8238.82	10,000	10,000	Diversifying
	1	507	0	0.51	0.348	3.113	0.0777	2737.956	8223.52	10,000	10,000	Diversifying
	1	520	0	1.32	0.912	3.348	0.0673	7186.682	7853.93	10,000	10,000	Diversifying
	1	540	0	0.848	0.463	3.789	0.0516	3649.878	6290.65	10,000	10,000	Diversifying
	1	564	0	2.609	2.121	4.585	0.0323	16,709.18	8368.46	10,000	10,000	Diversifying
	1	569	0	0.929	0.687	3.11	0.0778	5412.893	6683.39	10,000	10,000	Diversifying
	1	647	0	1.242	0.669	3.163	0.0753	5271.024	7073.87	10,000	10,000	Diversifying
	1	655	0	1.358	0.752	3.028	0.0818	5925.778	6323.58	10,000	10,000	Diversifying
	1	660	0	1.352	0.752	3.326	0.0682	5921.829	6786.94	10,000	10,000	Diversifying
GLUL	1	56	0	0.453	0.354	0.893	0.3445	0.771	3826.94	10,000	10,000	Diversifying
	1	59	0	1.65	1.246	1.022	0.3121	2.71	6186.49	10,000	10,000	Diversifying
	1	122	0	0.278	0.159	1.052	0.305	0.345	1464.52	10,000	10,000	Diversifying
	1	123	0	0.298	0.198	0.731	0.3925	0.431	1464.53	10,000	10,000	Diversifying
	1	148	0	0.278	0.162	1.068	0.3013	0.353	1464.56	10,000	10,000	Diversifying
	1	165	0	0.63	0.475	1.102	0.2938	1.033	3827.09	10,000	10,000	Diversifying
	1	300	0	0.581	0.431	1.23	0.2673	0.937	3827.10	10,000	10,000	Diversifying
	1	305	0	1.357	0.961	1.861	0.1725	2.089	6810.15	10,000	10,000	Diversifying

**Figure 3:**
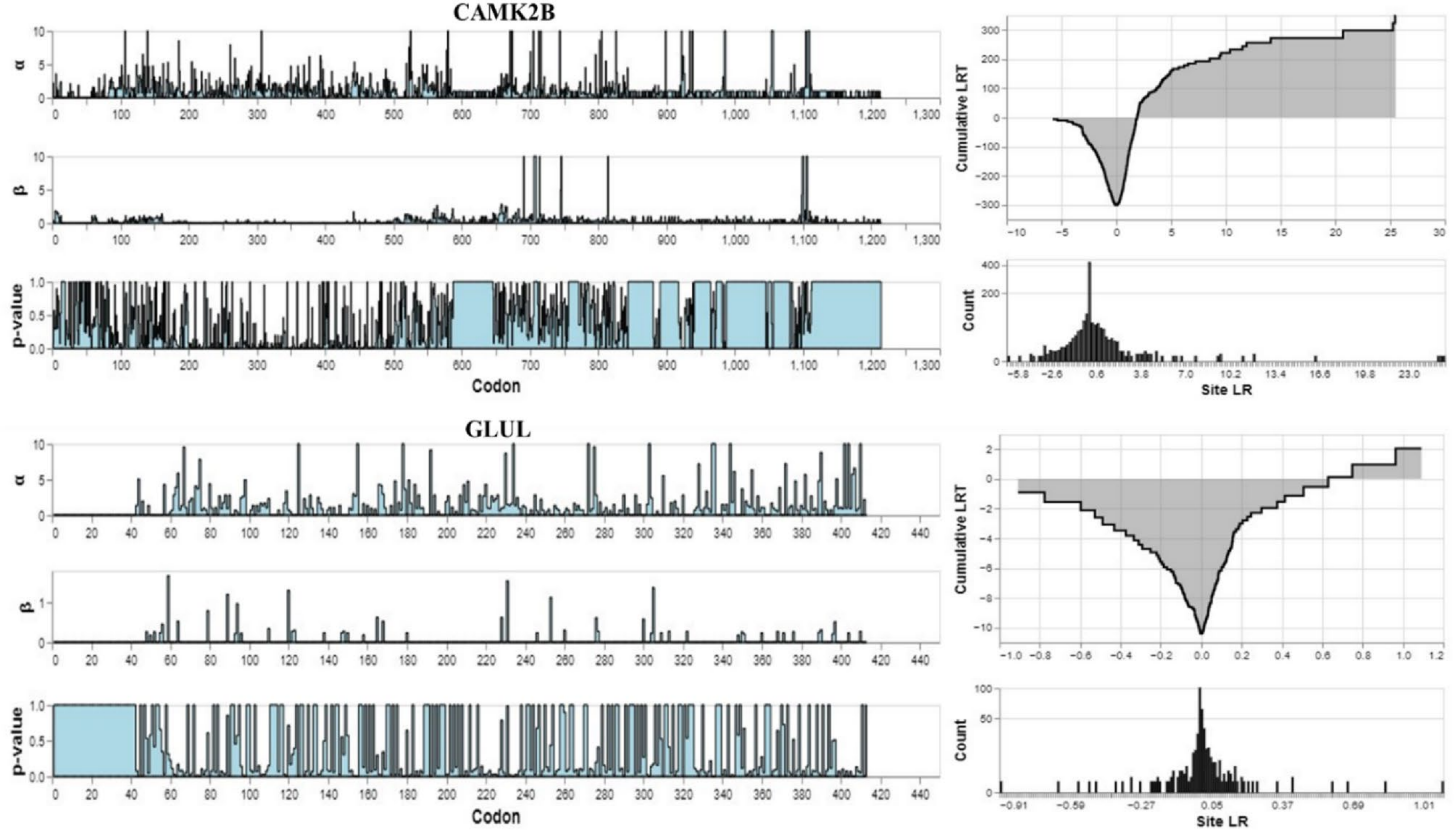
Cumulative distribution of the likelihood ratio test for the BUSTED test broken down by the contributions of individual sites.

“Episodic diversifying selection” refers to the phenomenon in which positive selection acts intermittently on specific locations across numerous lineages or branches of a phylogenetic tree, resulting in genetic diversification. According to our analysis, 43 sites within the CAMK2B gene may have undergone positive selection at some point during evolution, indicating their potential functional or adaptive significance (Fig. 3). Further research and characterization of these sites could offer valuable insights into the molecular mechanisms driving evolutionary changes within the dataset.

### Evolutionary fingerprinting analysis

**Figure 4:**
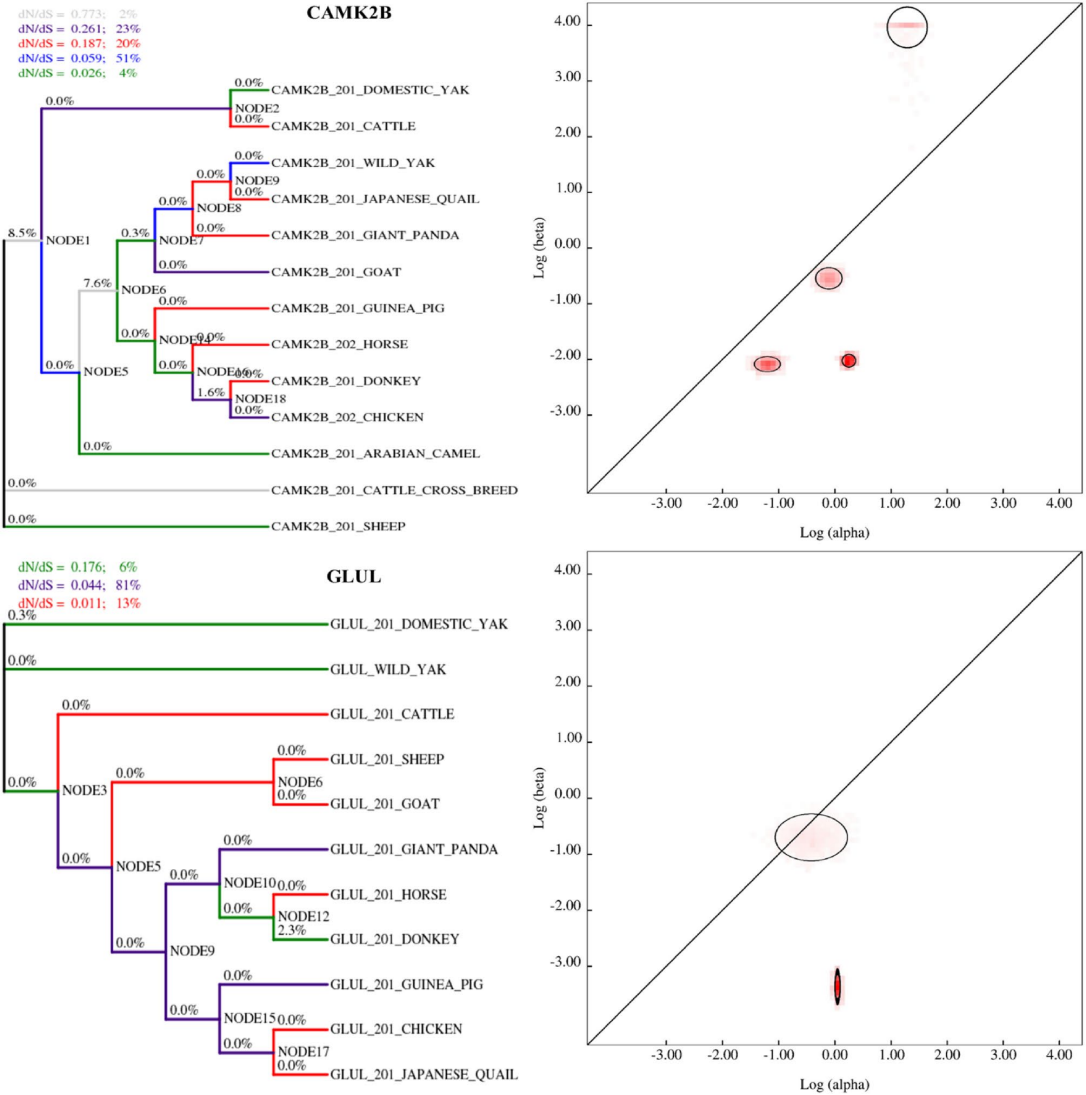
Phylogenetic analysis of synonymous and non-synonymous variant ratios in yak CAMK2B and GLUL genes**.**. This figure presents a phylogenetic analysis of the CAMK2B and GLUL genes in the yak, focusing on the ratio of synonymous (silent) to non-synonymous (amino acid-altering) variants. Displayed on a logarithmic scale, the plot provides an estimated distribution of these ratios as inferred from the gene alignment. Each ellipse approximates the Gaussian variance around the rate estimates, with the color gradient representing the density of posterior samples within the distribution for each rate estimate. Points located above the diagonal line indicate positive selection (ω > 1), suggesting adaptive evolutionary changes. In contrast, points below the line signify negative or purifying selection (ω < 1), indicating the removal of disadvantageous variants. The diagonal line itself represents the threshold of neutral evolution (ω = 1), where the rates of synonymous and non-synonymous variants are equal, implying no selection pressure**.**

Domesticated yaks (*B. grunniens*) have remarkable adaptations for life at high altitudes in the Himalayan region, a hallmark of which is their efficient nutrient assimilation from typically low-quality forage. Our analyses reveal that these yaks have undergone adaptive evolution in genes associated with nutrition pathways, significantly enhancing their ability to assimilate nutrients in oxygen-thin high-altitude environments. In particular, genes involved in the metabolism of carbohydrates and lipids, as well as those crucial for nutrient absorption and transport, show signs of positive selection in yaks. This is in contrast to their low-altitude counterparts, such as cattle. For instance, the CAMK2B and GLUL genes, which encode for key components in calcium signaling and nitrogen metabolism, respectively, have been positively selected in yaks (Fig. 4). This finding underscores their pivotal role in the physiological adaptations that allow yaks to prosper in the challenging conditions of high altitudes. We have identified several unique amino acid changes in the CAMK2B and GLUL genes of yaks compared to cattle, which could confer alterations in the structure and functionality of their respective proteins. Such changes are likely to influence the enzymes’ activities and regulatory mechanisms, underlining the notion that yaks' remarkable high-altitude adaptability may stem from the adaptive evolution of these critical genes. To further elucidate the selective forces at play, we performed phylogenetic tree analysis utilizing the BUSTED method, which detects positive selection across different nodes of the tree. By evaluating the ratio of synonymous to non-synonymous changes at each site, our analysis aimed to pinpoint sites under positive selection (Fig. 4). Considering three categories of non-synonymous rate variation across 59 median branches/partitions, our likelihood ratio test uncovered evidence of episodic diversifying selection within the dataset (Table 2).

“Episodic diversifying selection” is characterized by sporadic bouts of positive selection at specific sites across a phylogenetic tree, leading to genetic variation. Our research indicates that up to 43 sites within the CAMK2B gene may have been subject to positive selection during yak evolution, which suggests their crucial role in functional or adaptive processes (see Fig. 3 for site-specific details). Further investigation into these sites promises to shed light on the evolutionary pressures and molecular mechanisms that have shaped yak genetics, enhancing our understanding of adaptation to high-altitude environments.

### Recombination analysis

To assess the genetic diversity and potential adaptation mechanisms of yaks, we performed recombination analysis on the CAMK2B and GLUL genes using the Genetic Algorithm for Recombination Detection (GARD) program. GARD systematically scanned the sequence alignments of these genes to detect segments indicative of past recombination events, which are crucial for introducing genetic variation.

**Figure 5:**
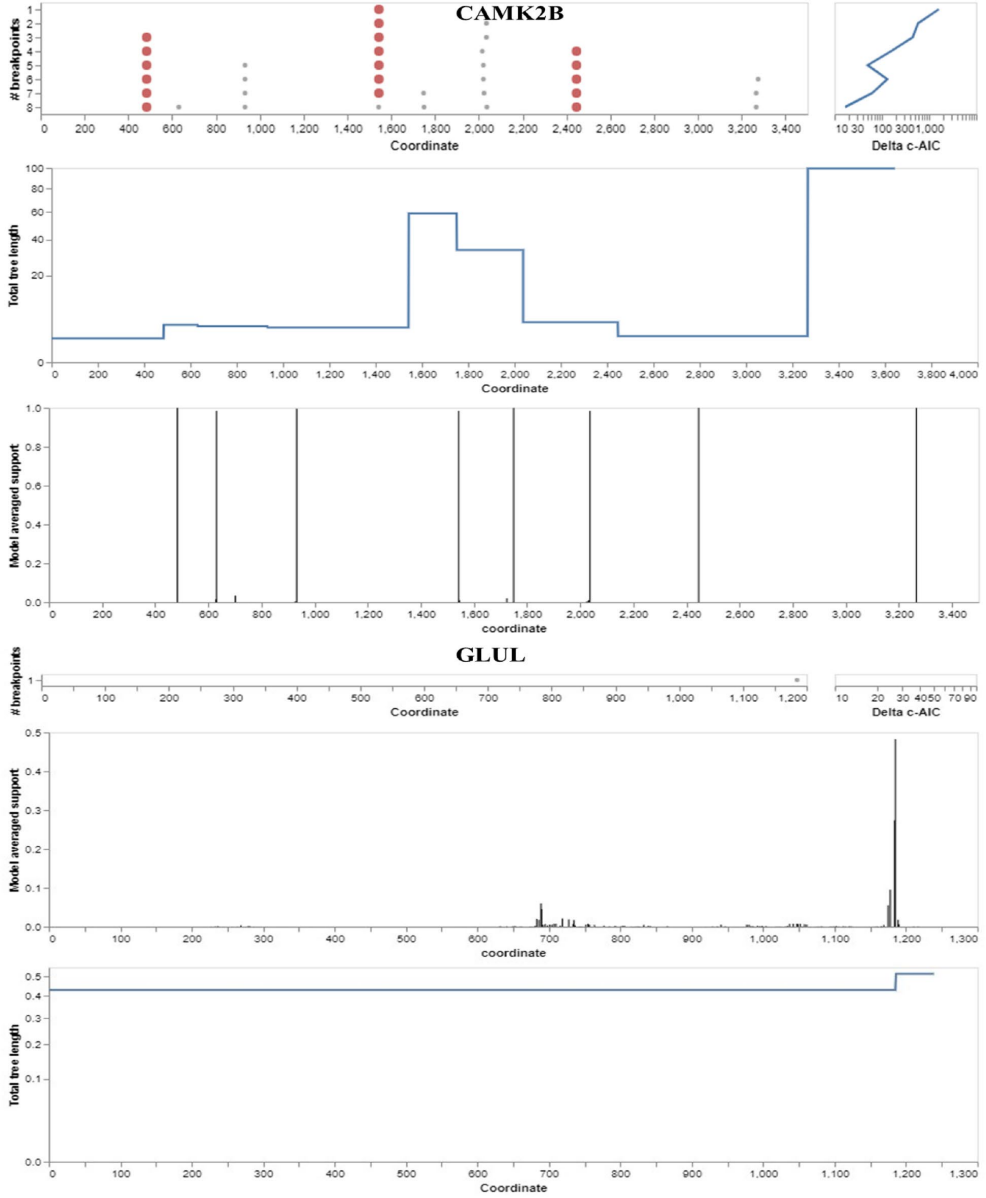
Genetic recombination of Yak genes showing evolutionary breakpoints in the genome. Left: the algorithm's maximum breakpoint location for each number of breakpoints considered. Right: the log scale c-AIC score rises between successive breakpoint values. Support for breakpoint placement based on an average model length of the entire tree by partition.

Our analysis was specifically aimed at identifying recombination breakpoints within these genes, suggesting that they may have experienced recombination throughout the evolutionary history of yaks. This methodological approach processed the data at a rate of 4.30 models per second, reviewing a total of 16,515 models. The sequence alignment indicated 3524 potential recombination breakpoints, creating an extensive search space of over 4.5 quadrillion models with up to 5 breakpoints possible within these genes (Fig. 5).

The detection of these recombination breakpoints in the yak's CAMK2B and GLUL genes points to possible genetic variations that could be influential in the phenotypic expression of these animals. The CAMK2B gene, which codes for a protein kinase involved in numerous physiological processes, including synaptic plasticity and learning, and the GLUL gene, responsible for glutamine synthesis, are both integral to glutamine metabolism. The occurrence of recombination within these genes could lead to variations in protein functionality or expression patterns, thereby influencing critical biological processes such as learning and memory, hormone metabolism, and amino acid synthesis. The impact of these recombination events is contingent upon the precise breakpoints and the resultant genetic changes.

Ongoing research into these recombination events and their impact on the yak's phenotype will deepen our understanding of the genetic foundations of specific traits, particularly those related to cognitive functions, hormonal regulation, and metabolism. This information could prove invaluable for selective breeding strategies aimed at enhancing certain traits within yak populations, thereby optimizing their adaptability and productivity in harsh high-altitude environments.

### Functional analysis

The Short-Chain Dehydrogenases/Reductases (SDR) family encompasses a broad range of enzymes that typically function as NAD- or NADP-dependent oxidoreductases. Characteristically, dehydrogenases within this family are structured with at least two domains: the first for coenzyme binding, usually NAD, and the second for substrate recognition and binding.

In our study, we focused on glutamine synthetase, an enzyme ubiquitous across all domains of life and pivotal in nitrogen metabolism, where it facilitates the assimilation of ammonium into glutamate. This enzyme’s functionality is essential for the yak's ability to manage nitrogen at high altitudes, where efficiency in nitrogen utilization is a key adaptive trait (Tables S3 and S4).

**Figure 6:**
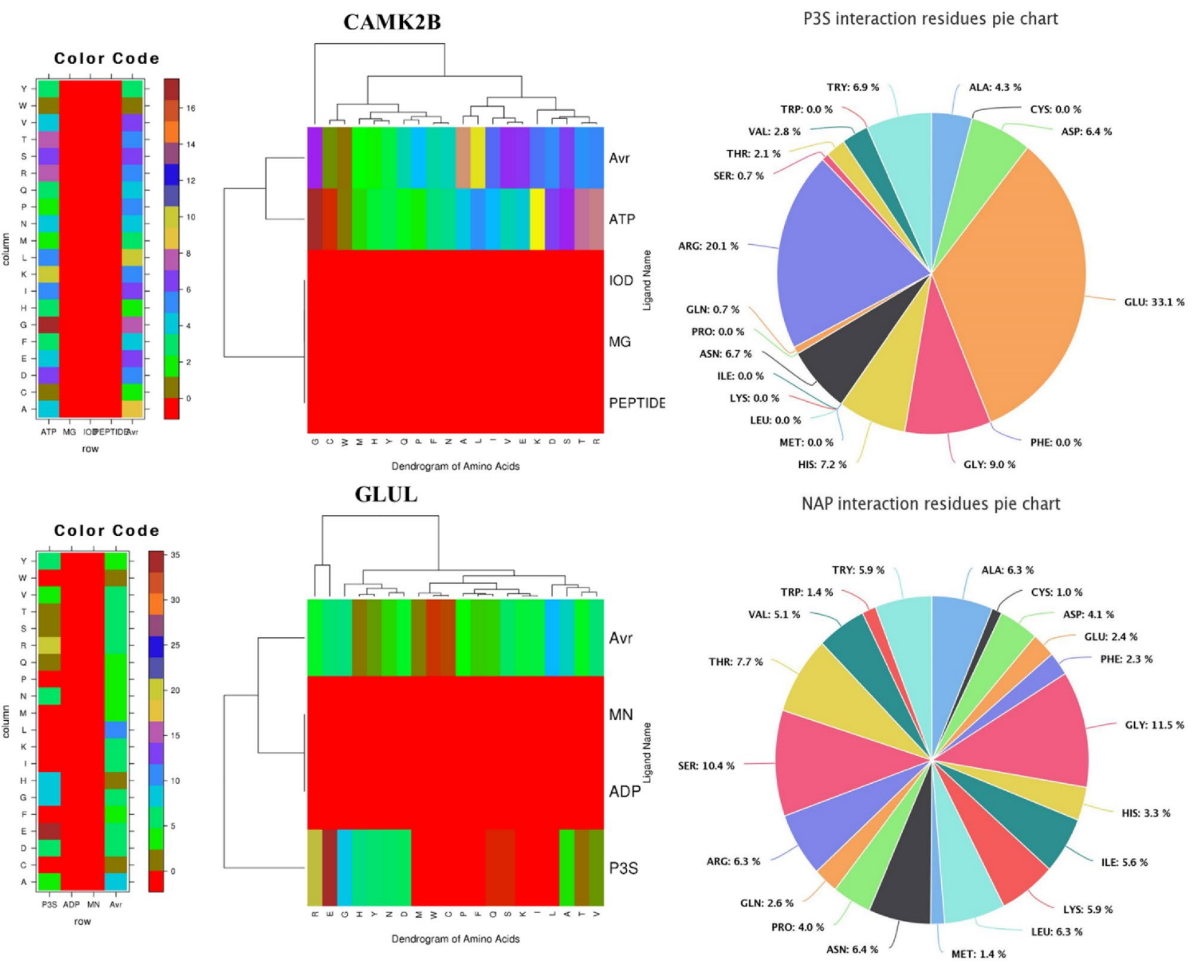
The clustering of amino acid residues that interact with one another. The structure of various ligand-binding residues is shown on the left, and the clustering of amino acids according to the physicochemical properties of ligand-interacting residues is shown on the right.

To delve into ligand binding interactions, which are contingent on the physiochemical properties of amino acids, we utilized a dedicated web server (http://crdd.osdd.net/raghava/lpicom). Our investigation revealed that the CAMK2B protein interacts with five distinct ligands, whereas the GLUL protein interacts with four. Notable among the CAMK2B ligands are ATP, IOD, MD, and PEPTIDE, and for GLUL, Mn, ADP, and P3S. These ligands engage with crucial amino acid residues spanning a diverse set, including alanine, cysteine, aspartic acid, glycine, histidine, lysine, leucine, arginine, serine, threonine, tryptophan, tyrosine, and valine (Fig. 6). We found that charged residues, particularly basic ones, are predominantly engaged in these interactions, which may be indicative of the high-energy demands at high altitudes.

For protein kinases, the serine/threonine and tyrosine kinases share a conserved catalytic core. Our analysis pinpointed two specific regions within this domain. The first, near the N-terminal, features a glycine-rich sequence adjacent to a lysine residue essential for ATP binding and is characterized by signature patterns unique to serine/threonine and tyrosine kinases. The central part of the domain harbors a conserved aspartic acid, indispensable for catalytic function (Fig. 6). These findings underscore the potential adaptations in kinase activity that are critical for energy regulation in yaks (Tables S3 and S4).

**Figure 7:**
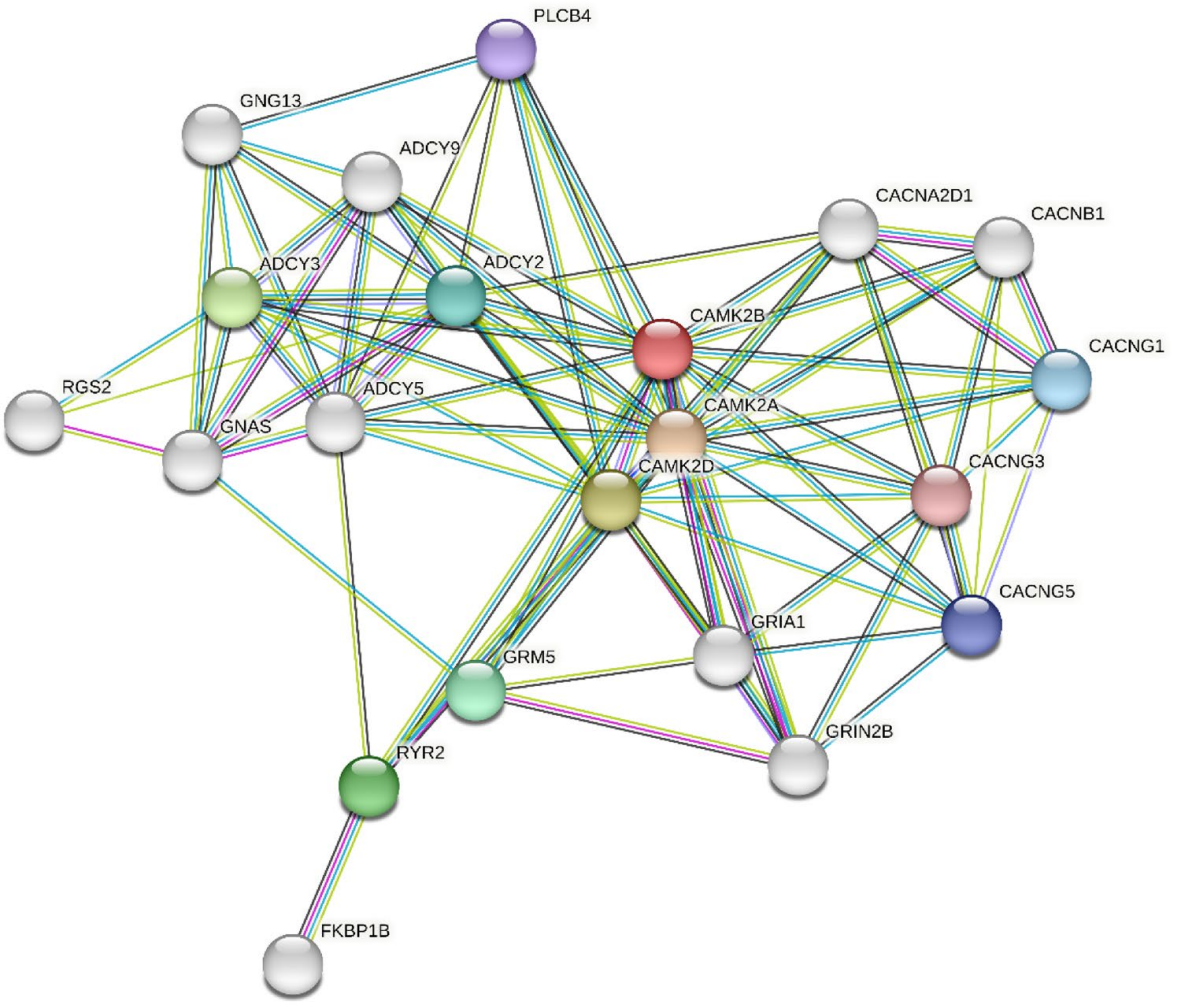
Analysis of protein–protein interactions in nutritional pathways. This figure visualizes the network of protein–protein interactions among proteins involved in the yak's nutritional pathways. The nodes, represented by circles, correspond to proteins; green nodes are proteins with well-characterized functions, while red nodes indicate proteins whose functions have not been fully elucidated. The black lines, or edges, connecting the nodes represent the interactions between proteins. The length of each line suggests the relative interaction distance or functional relationship strength between the proteins—shorter lines imply a closer or more direct interaction. This network provides insights into the complex interplay of proteins that govern nutritional processes, highlighting both known and potentially novel interactions that contribute to the yak's high-altitude adaptability.

Leveraging the STRING database, we explored protein interactions within networks pertinent to food metabolism, oxygen transport, energy production, and thermoregulation—key processes for high-altitude survival. We identified pathways involved in oxygen transport, hypoxic response, and energy metabolism, such as the AMPK and HIF-1 pathways, along with hemoglobin-related genes (Fig. 7). These pathways and their interactions shed light on the intricate genetic adaptations of yaks to their hypoxic and nutrient-challenged environments, offering deeper insights into the molecular basis of their remarkable high-altitude adaptability.

The interactions among nutritional pathway genes in yaks and the associated protein–protein interactions highlighted by our study provide a valuable framework for understanding nutrient assimilation and metabolic adaptation at high altitudes. These insights have broader implications, potentially informing conservation efforts, livestock management, and medical research into hypoxia-related conditions.

### Transcriptome expression

**Figure 8:**
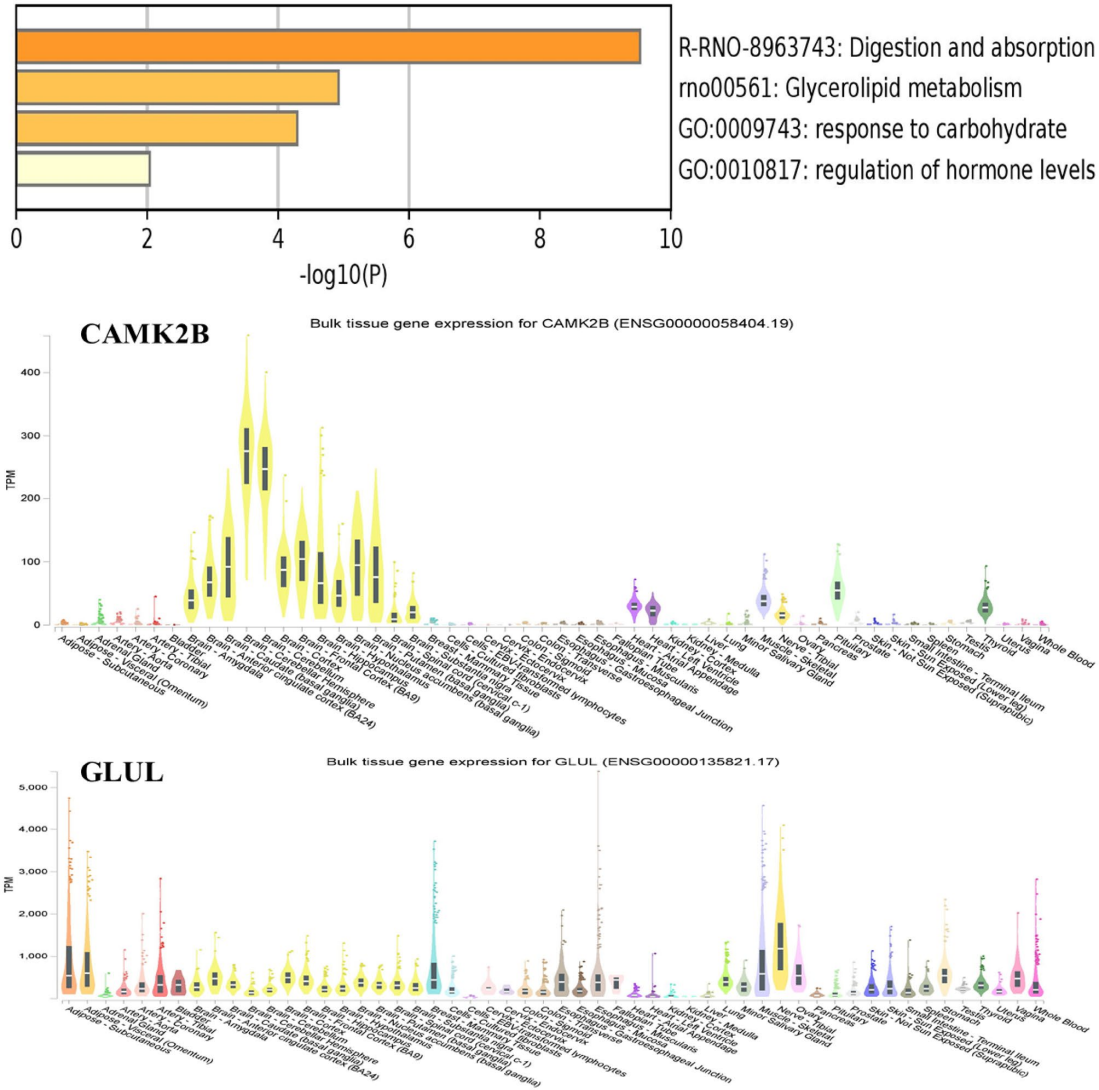
Tissue-specific expression patterns of nutritional pathway genes in yak. This figure displays the differential expression of genes involved in the yak's nutritional pathways across a range of tissue types, with data sourced from the GTEx consortium. The graphical representation illustrates the relative expression levels of these genes, highlighting tissues where each gene is most actively transcribed. This tissue-specific expression profile provides insights into the functional roles of these genes in different biological processes and systems within the yak. Understanding these patterns is essential for elucidating the genetic mechanisms that enable yaks to adapt and thrive in the demanding high-altitude environments where they reside.

The GTEx database, through its expression quantitative trait locus (eQTL) browser, offers an invaluable visualization of the intricate relationship between genetic variation and tissue-specific expression phenotypes. This browser encapsulates data from a comprehensive national research project that seeks to elucidate the associations between genetic polymorphisms and expression patterns on a high-throughput scale. Notably, it reveals that a diverse array of genes is implicated in multiple tissue types. Our exploration within this repository highlighted that the CAMK2B gene is predominantly expressed in the brain, heart, pituitary, and skeletal muscles, suggesting its involvement in critical functions related to these tissues. Conversely, GLUL is expressed in a distinct set of tissues, including arteries, adipose tissue, the colon, and skin (Fig. 8). These expression patterns underscore the varied roles these genes play in different physiological processes.

To gauge the average expression across a significant cohort of genes, we employed various enrichment analyses. These analyses provided a spectrum of results that were not uniformly indicative of tissue enrichment related to specific diseases or well-defined biological functions. Such variability in gene expression patterns across tissues hints at a complex interplay of genetic factors, which a myriad of environmental and physiological contexts may influence. These contexts likely exert nuanced effects on the regulatory mechanisms governing gene expression.

The data gleaned from the GTEx database, particularly concerning the expression of genes like CAMK2B and GLUL, reinforces the concept that gene regulation and expression are context-dependent and multifaceted. The associations between these expression profiles and particular tissue functions or disease states are complex and warrant further investigation to unravel the regulatory networks at play.

## Discussion

Positive selection is a critical evolutionary mechanism driving gene adaptation across species, including the yak (*B. grunniens*). For yaks residing at high altitudes, where resources are scarce, the adaptive evolution of genes within nutritional pathways is essential for their survival and reproductive success. Such environments, characterized by low oxygen levels, extreme temperatures, and limited food supplies, impose selective pressures that favor genetic variations, enhancing nutrient utilization and energy metabolism^24^. Positive selection influences yaks’ adaptive evolution through several mechanisms. It can alter coding regions of genes, leading to amino acid changes that enhance enzyme efficiency or specificity. For instance, adaptations in carbohydrate metabolism genes, such as those encoding amylase or glucose transporters, may improve the breakdown of complex carbohydrates into usable energy^25^. Additionally, positive selection may modify regulatory elements, affecting gene expression in response to the challenges posed by high altitudes^26^. This could manifest as the increased expression of genes facilitating nutrient assimilation or reduced expression of those less critical in such environments.

Moreover, positive selection may act on genes involved in nutrient sensing and signaling pathways, giving an advantage to yaks that can efficiently detect and respond to nutrient availability, optimizing energy use and nutrient uptake under resource constraints^27^. While ongoing research continues to explore the specific genes under positive selection in high-altitude yaks^28^, current studies suggest that genes related to energy metabolism, digestion, and nutrient transport may be under positive selection. Such insights not only illuminate the genetic underpinnings of yak adaptability to high altitudes but also have broader implications for our understanding of how organisms adapt to extreme conditions, aiding conservation efforts and informing livestock breeding programs.

Positive selection extends beyond nutritional pathways to include genes associated with lipid metabolism, such as APOA4 and APOB. These genes play a role in lipid transport and may enhance yaks' ability to use fatty acids as an energy source^28^. Additionally, positive selection on the LIPF gene, which encodes lipase, suggests yaks may efficiently digest and absorb dietary fatty acids, which are vital for energy production and metabolic processes.

Our study has also identified regions within the CAMK2B gene exhibiting signs of positive selection through FEL analysis, indicating that changes in calcium signaling pathways may be integral to yaks' high-altitude adaptability. Similarly, positive selection affecting steroid hormone and bile acid metabolism genes may contribute to their dietary adaptability at high altitudes^29^. Moreover, the positive selection observed at GLUL gene sites could reflect adaptations in amino acid availability, a critical factor in high-altitude environments^30^. The activation of particular biochemical pathways or gene networks that control external stimuli is indicated by gene expression patterns, which also shed light on the function of regulatory variation in adaptive evolution^31^. Different genes and pathways that are extensively involved in a variety of biological processes, including adaptations to hypoxia, have been found in recent investigations. A particular set of transcription factors known as the “master regulator” of O2 homeostasis mediates changes in gene expression during times of decreased O2 availability. Hypoxia-inducible factors (HIF) are the collective term for these genes^32^. Hypoxia-inducible factors are oxygen-dependent transcriptional activators that play a critical role in the development of mammals and tumor angiogenesis by regulating the transcription of genes related to oxygen homeostasis in response to hypoxia^33^. The most thoroughly studied and comprehended fundamental mediators of cellular adaptation to hypoxia in mammalian cells are the HIFα isoforms (HIF-1α and HIF-2α). HIF1α regulates the expression of hundreds of genes, acting as a crucial regulator of O_2_ homeostasis that synchronizes oxygen sensing and intracellular responses to hypoxia. A subset of the HIF1α-regulated genes are involved in the metabolic processes of energy, angiogenesis, erythropoiesis, iron homeostasis, and apoptosis^28^.

Yaks should be a model animal for comprehending the molecular basis of high-altitude adaptation due to their outstanding physical endurance and adaptability to high-altitude conditions. Optimizing animal productivity, especially in situations where feed is scarce, is based on the effective conversion and utilization of nutrients. Yaks exhibit “low-carbon” and “nitrogen-saving” features, which are indicative of a higher nutritional utilization than cattle after long-term adaption. Because of these adaptations, yaks are able to adapt to the varying seasonal availability of fodder, particularly during the lengthy winter months when dietary intakes are incredibly low.

Understanding these genetic adaptations contributes to our knowledge of how yaks and potentially other species, including humans, adapt to high-altitude conditions. The ability of yaks to efficiently utilize nutrients from low-quality fodder in such hostile environments underscores the importance of studying these adaptive genetic changes. By identifying positively selected genes within nutrition pathways, we gain a window into the physiological mechanisms supporting yaks' successful habitation of high altitudes, providing a template for further research into evolutionary adaptation strategies.

It is crucial to note that the precise findings and implications of this study would be clearer with access to the complete research article. Nonetheless, examining the positive selection of genes within nutritional pathways for high-altitude adaptation in yaks is a compelling avenue of research that can deepen our understanding of evolutionary processes in harsh environments.

## Conclusions

Our study elucidates the pivotal role of positive selection in the adaptive evolution of nutrition pathway genes in the yak. These species have become emblematic of successful high-altitude adaptation. Indigenous to the Tibetan Plateau, yaks have evolved to navigate the challenges of their lofty habitats, where thin air and scarce resources prevail. Through comprehensive comparative genomic and molecular evolutionary analyses, we have discovered a notable enrichment of genes within the yak's dietary pathway that have undergone positive selection. These genes orchestrate critical functions related to the metabolism of carbohydrates, lipids, proteins, and the digestion of vitamins and minerals. The positive selection of these genes likely confers enhanced nutrient assimilation efficiency, facilitating metabolic adjustments necessary for survival in oxygen-deprived high-altitude environments. Our findings particularly underscore the enhanced selection pressure on energy metabolism genes in yaks, highlighting the adaptive importance of efficient energy management in low-oxygen conditions. The occurrence of parallel positive selection patterns in genes of other high-altitude species points to a broader evolutionary phenomenon of convergent evolution, where distinct species independently acquire similar genetic adaptations in response to equivalent environmental challenges. By pinpointing genes under positive selection related to the yak's nutrition pathways, our research offers a deeper understanding of the molecular strategies that underpin this species' extraordinary capacity to thrive in an environment where nutrients are at a premium. This knowledge not only advances our grasp of evolutionary processes in high-altitude adaptation but also has wider ramifications for the study of other high-altitude fauna. The insights garnered from this research contribute to the field of evolutionary biology by providing a clearer picture of how natural selection shapes the genetic toolkit of species for optimal nutrient utilization in demanding ecosystems. Furthermore, the implications of our work extend beyond yaks, potentially informing conservation strategies and the management of other species that inhabit extreme altitudes, as well as shedding light on the genetic and molecular bases of human adaptation to high-altitude living.

## Materials and methods

### Sequence analysis

The whole genome yak (*B. grunniens*) sequences were retrieved from the Ensembl database. Specifically, we focused on the amino acid and nucleotide sequences of nutrition pathway genes CAMK2B (ENSBMUG00000026255) and GLUL (ENSBMUG00000026918), which play a crucial role in nutritional absorption at high elevations and are expressed in yaks compared with other mammalian species (Supplementary file). Coding sequences for CAMK2B were obtained from Ensembl, covering various mammalian species based on gene annotation, which considered two conserved neighboring genes^34^. The BLASTn v2.2.29 + algorithm was used to choose the most suitable scaffold^35^. Annotation procedures included confirming the presence of start and stop codons, which were determined using MITOS^36^. We conducted alignments for protein-coding and ribosomal genes using MACSE v1.01b^37^ and ClustalW v2^38^. Genes that were less than one-third the length of the overall locus alignment were excluded from further analysis.

### Phylogenetic analysis

Gene sequences from Yak CAMK2B and GLUL were employed to construct phylogenetic trees, elucidating the evolutionary relationships and changes in these genes over time. Phylogenetic trees were generated using MEGA (Molecular Evolutionary Genetics Analysis) version 10.0.5^39^ using a maximum likelihood method. The topology of the tree constructed using the neighbor-joining method was assessed by applying the maximum likelihood method and the Whelan and Goldman (WAG) substitution model^40^. To ensure the robustness of the tree structure, we performed 1000 bootstrap repetitions. For precise comparisons and evaluations of gene trees and other phylogenetic information, we used the TreeBeST-generated species tree as a reference (http://treesoft.svn.sourceforge.net/viewrc/treesoft/).

### Selection analyses

To assess the presence of selective pressure on homologous nutritional pathway genes, we compared the ratio of synonymous to non-synonymous substitutions (dN/dS). We calculated ω using the PAML codon-based maximum likelihood approach, CODEML. Two different PAML models were employed to determine variations in selective pressures across different grasshopper lineages. Our analysis focused on ω values at the terminations of branches, assessing the rate of mutations accumulated between modern species and their closest reconstructed relatives. In line with the free-ratio model^41^, the ω values at each branch were assumed to be random. Initial detection of positive selection was carried out using the branch-site model in PAML ^42^, where the null hypothesis set ω = 1. Statistical significance levels were determined using a chi-square distribution based on the difference in the number of parameters between the two models, twice the difference in log-likelihood values, and degrees of freedom. The identification of positive selection can sometimes vary due to disparities in methods, assumptions, methodologies, and gene conversion bias among different approaches^43^. Bonferroni's correction was applied for multiple tests in the PAML site-branch model with various parameters. We validated our findings using independent tools, including the HyPhy package^44^. Site models (M1, M2, M8a, and M8) were used to allow ω to vary between sites, estimating the likelihood of each site within each gene being a target of positive selection. This model assessed the likelihood of positive selection signals occurring at a limited number of sites during relatively short evolutionary periods. In the branch-site test, both the alternative model of positive selection (ω > 1) and the null model of neutral evolution (ω = 1) were employed to determine whether each branch experienced selective pressure. The alternative model predicted higher selection levels on each branch compared to the null model. This methodology was applied to locate instances of positive selection at a limited number of genomic loci across all grasshopper lineages. The likelihood ratio test (LRT) was employed to evaluate and select the most suitable model for our data.

### Protein domain and structure analysis

In this stage, sites identified as positively selected were subjected to further structural analysis. The PSIPRED 4.0 program was employed for predicting protein secondary structures^45^ and the AlphaFold2 database was utilized to infer the tertiary structures of Yak proteins^46^. For the prediction of specific sites for kinase phosphorylation and binding domains, SCANSITE 4.0 was used, drawing on a database of 81 mammalian kinases/domains^47^. The output from SCANSITE was filtered at a “high” rigor level, followed by a manual examination of linker regions and domains. To better understand the functional significance of the sites under positive selection, we overlaid them on the 3D structures of the proteins. Homology modeling for predicting 3D gene structures was performed using the I-TASSER server, with input from the Yak genome available in GenBank^48^. Additional functional information about the genes believed to be under positive selection was sourced from UniProt^49^.

### Functional analysis

For protein sequence analysis, two online tools were utilized: Clustal W for sequence alignment and the LPIcom server for annotating amino acid similarities. Identified proteins were categorized according to their placement within specific gene ontology (GO) hierarchy levels, with an emphasis on the “Biological Process” (GOBP) class. This categorization utilized the “groupGO” function. Enrichment tests for GOBP keywords were conducted using the "enrichGO" function, comparing the distribution of protein kinases to a background list of all proteins in the relevant annotation database. Visualization of all GO terms related to nutrient metabolism was facilitated using the g: GOSt web tool within the g: Profiler suite^50^, in conjunction with Cytoscape’s Enrichment Map program^51^. Data from large-scale transcriptome studies were integrated with information from the Genotype-Tissue Expression (GTEx) database Release V8 (dbGaP Accession phs000424.v8.p2)^52^, providing insights into gene-level associations and explaining how gene expression levels influence phenotypes. To further understand the biology of CAMK2B and GLUL genes, we searched for genes associated with activating transcription factors using the bulk tissue expression panel, incorporating the term “activating transcription factor” alongside gene information for CAMK2B (ENSG00000058404.19) and GLUL (ENSG00000135821.17). Tissue samples were sourced from 54 healthy sites across nearly 1000 individuals^52^.

### Supplementary Information

Supplementary Tables.

## Data Availability

All data relevant to this article shall be available from the corresponding author.
