# Author Correction: Genomic insights into Yak (*Bos grunniens*) adaptations for nutrient assimilation in high-altitudes

**DOI:** 10.1038/s41598-024-68548-8

**Published:** 2024-08-05

**Authors:** Hafiz Ishfaq Ahmad, Sammina Mahmood, Mubashar Hassan, Muhammad Sajid, Irfan Ahmed, Borhan Shokrollahi, Abid Hussain Shahzad, Shaista Abbas, Sanan Raza, Khan Komal, Sayyed Aun Muhammad, Dalia Fouad, Farid S. Ataya, Zhengtian Li

**Affiliations:** 1 Department of Animal Breeding and Genetics, Faculty of Veterinary and Animal Sciences, The Islamia University of Bahawalpur, Bahawalpur, Pakistan; 2 Department of Botany, Division of Science and Technology, University of Education, Lahore, Pakistan; 3 Department of Clinical Sciences, College of Veterinary and Animal Sciences (Sub Campus UVAS, Lahore), Jhang, 35200 Pakistan; 4 Department of Pathobiology, College of Veterinary and Animal Sciences (Sub Campus UVAS, Lahore), Jhang, 35200 Pakistan; 5 Department of Animal Nutrition, Faculty of Veterinary and Animal Sciences, The Islamia University of Bahawalpur, Bahawalpur, Pakistan; 6 Hanwoo Research Institute, National Institute of Animal Science, Pyeongchang, 25340 Korea; 7 Department of Physiology and Biochemistry, College of Veterinary and Animal Sciences, Jhang, 35200 Pakistan; 8 Department of Basic Sciences, Anatomy Section, College of Veterinary and Animal Sciences, Jhang, 35200 Pakistan; 9 Department of Zoology, College of Science, King Saud University, PO Box 22452, 11495 Riyadh, Saudi Arabia; 10 Department of Biochemistry, College of Science, King Saud University, PO Box 2455, 11495 Riyadh, Saudi Arabia; 11 College of Biological Resource and Food Engineering, Qujing Normal University, Yunnan, 655011 China

Correction to: *Scientific Reports* 10.1038/s41598-024-55712-3, published online 07 March 2024

The original version of this Article contained errors.

Borhan Shokrollahi was incorrectly affiliated with ‘Department of Animal Science, Sanandaj Branch, Islamic Azad University, Sanandaj, Kurdistan, Iran’. The correct affiliation is listed below.

Hanwoo Research Institute, National Institute of Animal Science, Pyeongchang, 25340, Korea

In addition, the Article contained an error in the Funding section.

“This research was supported by Yunnan Fundamental Research Projects (202101AU070147), the Special Basic Cooperative Research Programs of Yunnan Provincial Undergraduate Universities’ Association (202101BA070001-210,202101BA070001-021), the Scientific Research Foundation of Yunnan Provincial Department of Education (2023J1037), the Special Basic Cooperative Research Innovation Programs of Qujing Science and Technology Bureau & Qujing Normal University (KJLH2022YB06, KJLH2023ZD07). The authors extend their appreciation to Researchers Supporting Project number (RSPD2023R965), King Saud University, Riyadh, Saudi Arabia.”

now reads:

“This research was supported by Yunnan Fundamental Research Projects (202101AU070147), the Special Basic Cooperative Research Programs of Yunnan Provincial Undergraduate Universities’ Association (202101BA070001-210,202101BA070001-021), the Scientifc Research Foundation of Yunnan Provincial Department of Education (2023J1037), the Special Basic Cooperative Research Innovation Programs of Qujing Science and Technology Bureau & Qujing Normal University (KJLH2022YB06, KJLH2023ZD07). The authors extend their appreciation to Researchers Supporting Project number (RSPD2024R693), King Saud University, Riyadh, Saudi Arabia.”

The original Article has been corrected.

