# Supplementary material for: Genomic insights into Yak (Bos grunniens) adaptations for nutrient assimilation in high-altitudes
Source: Sci Rep. 2024 Mar 7;14:5650. doi: 10.1038/s41598-024-55712-3 (PMC10920680; doi:10.1038/s41598-024-55712-3)
Supplement: Supplementary file 1 — Supplementary Tables. [file 41598_2024_55712_MOESM1_ESM.docx]

**Table S1: List of mammalian species with accession number used for analysis of CAMK2B gene**

| **Gene ID** | **Scientific name** | **Common name** | | **Transcript accessions** | **Protein accessions** |
| --- | --- | --- | --- | --- | --- |
| 816 | *Homo sapiens* | human | | NM_001220.5 | NP_001211.3 |
| 12323 | *Mus musculus* | house mouse | | NM_007595.6 | NP_031621.3 |
| 24245 | *Rattus norvegicus* | Norway rat | | NM_021739.2 | NP_068507.2 |
| 482687 | *Canis lupus familiaris* | dog | | XM_038452392.1 | XP_038308320.1 |
| 525416 | *Bos taurus* | cattle | | XM_010804378.4 | XP_010802680.1 |
| 706977 | *Macaca mulatta* | Rhesus monkey | | XM_028845801.1 | XP_028701634.1 |
| 738167 | *Pan troglodytes* | chimpanzee | | XM_016957386.3 | XP_016812875.1 |
| 1E+08 | *Monodelphis domestica* | gray short-tailed opossum | | XM_001369990.4 | XP_001370027.2 |
| 1E+08 | *Ornithorhynchus anatinus* | platypus | | XM_029074790.2 | XP_028930623.1 |
| 1E+08 | *Equus caballus* | horse | | XM_023638990.1 | XP_023494758.1 |
| 1E+08 | *Oryctolagus cuniculus* | rabbit | | XM_051853040.1 | XP_051709000.1 |
| 1E+08 | *Callithrix jacchus* | white-tufted-ear marmoset | | XM_035253292.2 | XP_035109183.1 |
| 1E+08 | *Pongo abelii* | Sumatran orangutan | | XM_024249746.2 | XP_024105514.1 |
| 1E+08 | *Ailuropoda melanoleuca* | giant panda | | XM_034664942.1 | XP_034520833.1 |
| 1.01E+08 | *Sus scrofa* | pig | | XM_021078635.1 | XP_020934294.1 |
| 1.01E+08 | *Nomascus leucogenys* | white-cheeked gibbon | | XM_030797202.1 | XP_030653062.1 |
| 1.01E+08 | *Loxodonta africana* | African savanna elephant | | XM_010598294.2 | XP_010596596.1 |
| 1.01E+08 | *Cavia porcellus* | domestic guinea pig | | XM_013148228.2 | XP_013003682.1 |
| 1.01E+08 | *Cricetulus griseus* | Chinese hamster | | XM_027388178.2 | XP_027243979.1 |
| 1.01E+08 | *Sarcophilus harrisii* | Tasmanian devil | | XM_031951517.1 | XP_031807377.1 |
| 1.01E+08 | *Otolemur garnettii* | small-eared galago | | XM_012809974.1 | XP_012665428.1 |
| 1.01E+08 | *Pan paniscus* | pygmy chimpanzee | | XM_034964030.2 | XP_034819921.1 |
| 1.01E+08 | *Papio anubis* | olive baboon | | XM_003896045.5 | XP_003896094.1 |
| 1.01E+08 | *Saimiri boliviensis* | Bolivian squirrel monkey | | XM_039462128.1 | XP_039318062.1 |
| 1.01E+08 | *Felis catus* | domestic cat | | XM_045052077.1 | XP_044908012.1 |
| 1.01E+08 | *Ovis aries* | sheep | | XM_027968714.3 | XP_027824515.1 |
| 1.01E+08 | *Gorilla gorilla gorilla* | western lowland gorilla | | XM_031013344.2 | XP_030869204.1 |
| 1.01E+08 | *Orcinus orca* | killer whale | | XM_049714677.1 | XP_049570634.1 |
| 1.05E+08 | *Camelus bactrianus* | Bactrian camel | | XM_045506347.1 | XP_045362303.1 |
| 1.05E+08 | *Camelus dromedarius* | Arabian camel | | XM_031454595.1 | XP_031310455.1 |
| 1.01E+08 | *simum* | southern white rhinoceros | | XM_014793608.1 | XP_014649094.1 |
| 1.01E+08 | *Dasypus novemcinctus* | nine-banded armadillo | | XM_058296685.1 | XP_058152668.1 |
| 1.02E+08 | *Ochotona princeps* | American pika | | XM_058678046.1 | XP_058534029.1 |
| 1.02E+08 | *Sorex araneus* | European shrew | | XM_055133954.1 | XP_054989929.1 |
| 1.02E+08 | *Octodon degus* | degu | | XM_023714417.1 | XP_023570185.1 |
| 1.22E+08 | *Panthera leo* | lion | | XM_042913150.1 | XP_042769084.1 |
| 1.02E+08 | *Condylura cristata* | star-nosed mole | | XM_012735159.1 | XP_012590613.1 |
| 1.03E+08 | *Vicugna pacos* | alpaca | | XM_031679815.1 | XP_031535675.1 |
| 1.02E+08 | *Mustela putorius furo* | domestic ferret | | XM_004739466.2 | XP_004739523.1 |
| 1.02E+08 | *Heterocephalus glaber* | naked mole-rat | | XM_004839914.2 | XP_004839971.1 |
| 1.02E+08 | *Mesocricetus auratus* | golden hamster | | XM_005086758.3 | XP_005086815.1 |
| 1.02E+08 | *Macaca fascicularis* | crab-eating macaque | | XM_045387688.1 | XP_045243623.1 |
| 1.02E+08 | *tridecemlineatus* | ground squirrel | | XM_005319238.4 | XP_005319295.1 |
| 1.05E+08 | *Bison bison* | | Bison | XM_010829115.1 | XP_010827417.1 |
| 1.02E+08 | *Chinchilla lanigera* | long-tailed chinchilla | | XM_013520515.1 | XP_013375969.1 |
| 1.02E+08 | *Capra hircus* | goat | | XM_018047014.1 | XP_017902503.1 |
| 1.02E+08 | *Myotis brandtii* | Brandt's bat | | XM_014530542.1 | XP_014386028.1 |
| 1.02E+08 | *Bos mutus* | wild yak | | XM_014479314.1 | XP_014334800.1 |

**Table S2: List of mammalian species with accession number used for analysis of GLUL gene**

| **Gene ID** | **Scientific name** | **Common name** | **Transcript accessions** | **Protein accessions** |
| --- | --- | --- | --- | --- |
| 2752 | *Homo sapiens* | human | NM_001033044.4 | NP_001028216.1 |
| 14645 | *Mus musculus* | house mouse | NM_008131.5 | NP_032157.2 |
| 24957 | *Rattus norvegicus* | Norway rat | NM_017073.4 | NP_058769.4 |
| 281199 | *Bos taurus* | cattle | NM_001040474.2 | NP_001035564.1 |
| 396944 | *Sus scrofa* | pig | XM_021102200.1 | XP_020957859.1 |
| 403443 | *Canis lupus familiaris* | dog | NM_001002965.3 | NP_001002965.2 |
| 456450 | *Pan troglodytes* | chimpanzee | NM_001279785.1 | NP_001266714.1 |
| 715952 | *Macaca mulatta* | Rhesus monkey | NM_001266013.2 | NP_001252942.1 |
| 1E+08 | *Equus caballus* | horse | XM_001489235.6 | XP_001489285.4 |
| 1E+08 | *Ornithorhynchus anatinus* | platypus | XM_029080333.2 | XP_028936166.1 |
| 1E+08 | *Oryctolagus cuniculus* | rabbit | XM_051840972.1 | XP_051696932.1 |
| 1E+08 | *Callithrix jacchus* | white-tufted-ear marmoset | XM_054247787.1 | XP_054103762.1 |
| 1E+08 | *Pongo abelii* | Sumatran orangutan | XM_002809716.5 | XP_002809762.1 |
| 1E+08 | *Ailuropoda melanoleuca* | giant panda | XM_019799817.2 | XP_019655376.1 |
| 1.01E+08 | *Nomascus leucogenys* | white-cheeked gibbon | XM_030818787.1 | XP_030674647.1 |
| 1.01E+08 | *Loxodonta africana* | African savanna elephant | XM_003410857.3 | XP_003410905.1 |
| 1.01E+08 | *Cavia porcellus* | domestic guinea pig | XM_003474859.4 | XP_003474907.1 |
| 1.01E+08 | *Cricetulus griseus* | Chinese hamster | XM_003502861.5 | XP_003502909.2 |
| 1.01E+08 | *Sarcophilus harrisii* | Tasmanian devil | XM_031936994.1 | XP_031792854.1 |
| 1.01E+08 | *Otolemur garnettii* | small-eared galago | XM_023510808.1 | XP_023366576.1 |
| 1.01E+08 | *Pan paniscus* | pygmy chimpanzee | XM_003824735.4 | XP_003824783.1 |
| 1.01E+08 | *Papio anubis* | olive baboon | XM_021929837.2 | XP_021785529.2 |
| 1.01E+08 | *Saimiri boliviensis* | Bolivian squirrel monkey | XM_039460105.1 | XP_039316039.1 |
| 1.01E+08 | *Felis catus* | domestic cat | XM_003999279.6 | XP_003999328.1 |
| 1.01E+08 | *Ovis aries* | sheep | XM_027975934.2 | XP_027831735.1 |
| 1.01E+08 | *Gorilla gorilla gorilla* | western lowland gorilla | XM_004028009.3 | XP_004028058.1 |
| 1.03E+08 | *Vicugna pacos* | alpaca | XM_006211784.3 | XP_006211846.2 |
| 1.01E+08 | *Tursiops truncatus* | common bottlenose dolphin | XM_019918426.2 | XP_019773985.1 |
| 1.01E+08 | *latirostris* | Florida manatee | XM_023728097.1 | XP_023583865.1 |
| 1.05E+08 | *Camelus bactrianus* | Bactrian camel | XM_010954936.2 | XP_010953238.1 |
| 1.05E+08 | *Camelus dromedarius* | Arabian camel | XM_031435642.1 | XP_031291502.1 |
| 1.01E+08 | *Dasypus novemcinctus* | nine-banded armadillo | XM_004469127.4 | XP_004469184.1 |
| 1.02E+08 | *Ochotona princeps* | American pika | XM_004578835.2 | XP_004578892.1 |
| 1.02E+08 | *Sorex araneus* | European shrew | XM_055121225.1 | XP_054977200.1 |
| 1.02E+08 | *Octodon degus* | degu | XM_023703600.1 | XP_023559368.1 |
| 1.02E+08 | *Jaculus jaculus* | lesser Egyptian jerboa | XM_045141982.1 | XP_044997917.1 |
| 1.02E+08 | *Condylura cristata* | star-nosed mole | XM_004688542.2 | XP_004688599.1 |
| 1.02E+08 | *Mustela putorius furo* | domestic ferret | XM_004767903.3 | XP_004767960.1 |
| 1.02E+08 | *Heterocephalus glaber* | naked mole-rat | XM_004853354.2 | XP_004853411.1 |
| 1.02E+08 | *Mesocricetus auratus* | golden hamster | XM_040738925.1 | XP_040594859.1 |
| 1.02E+08 | *Macaca fascicularis* | crab-eating macaque | XM_005540162.3 | XP_005540219.1 |
| 1.02E+08 | *Ictidomys tridecemlineatus* | thirteen-lined ground squirrel | XM_040278202.1 | XP_040134136.1 |
| 1.02E+08 | *Microtus ochrogaster* | prairie vole | XM_005363885.2 | XP_005363942.1 |
| 1.02E+08 | *Chinchilla lanigera* | long-tailed chinchilla | XM_005375000.2 | XP_005375057.1 |
| 1.02E+08 | *Capra hircus* | goat | XM_018060686.1 | XP_017916175.1 |
| 1.02E+08 | *Myotis brandtii* | Brandt's bat | XM_005880220.2 | XP_005880282.1 |
| 1.02E+08 | *Bos mutus* | wild yak | XM_005910126.2 | XP_005910188.1 |
| 1.02E+08 | *Bubalus bubalis* | water buffalo | XM_006048610.4 | XP_006048672.1 |

**Table S3: Functional analysis of CAMK2B and GLUL genes in the Yak genome, including pathways and biological processes**

| **GO** | **Category** | **Description** | **Count** | **%** | **Log10(P)** | **Log10(q)** |
| --- | --- | --- | --- | --- | --- | --- |
| hsa04261 | KEGG Pathway | Adrenergic signaling in cardiomyocytes | 10 | 55.56 | -18.54 | -14.38 |
| hsa05414 | KEGG Pathway | Dilated cardiomyopathy | 6 | 33.33 | -10.80 | -7.60 |
| hsa04972 | KEGG Pathway | Pancreatic secretion | 5 | 27.78 | -8.48 | -5.63 |
| GO:0051966 | GO Biological Processes | regulation of synaptic transmission, glutamatergic | 4 | 22.22 | -6.91 | -4.24 |
| GO:0016042 | GO Biological Processes | lipid catabolic process | 5 | 27.78 | -6.21 | -3.68 |
| GO:0006874 | GO Biological Processes | cellular calcium ion homeostasis | 4 | 22.22 | -5.36 | -2.99 |
| GO:0010817 | GO Biological Processes | regulation of hormone levels | 3 | 16.67 | -2.47 | -0.43 |

**Table S4: GO term analysis of Yak genes showing functional pathways**

| **Go term** | **Description** | **Network count** | **Strength** | **FDR** |
| --- | --- | --- | --- | --- |
| GO:0099583 | Cytosolic calcium ion concentration | 2 of 3 | 2.82 | 0.001 |
| GO:0004016 | Adenylate cyclase activity | 4 of 11 | 2.56 | 4.16E-07 |
| GO:0004683 | Calmodulin-dependent protein kinase activity | 3 of 18 | 2.22 | 0.00016 |
| GO:0005245 | Voltage-gated calcium channel activity | 5 of 37 | 2.13 | 3.42E-07 |
| GO:0099604 | Ligand-gated calcium channel activity | 2 of 15 | 2.12 | 0.011 |
| GO:0008066 | Glutamate receptor activity | 3 of 25 | 2.08 | 0.00035 |
| GO:0004970 | Ionotropic glutamate receptor activity | 2 of 18 | 2.04 | 0.0144 |
| GO:0005262 | Calcium channel activity | 7 of 101 | 1.84 | 2.50E-08 |
| GO:0035254 | Glutamate receptor binding | 2 of 34 | 1.77 | 0.0371 |
| GO:0005246 | Calcium channel regulator activity | 2 of 38 | 1.72 | 0.0448 |
| GO:0098960 | Postsynaptic neurotransmitter receptor activity | 3 of 62 | 1.68 | 0.0036 |
| GO:0022843 | Voltage-gated cation channel activity | 6 of 127 | 1.67 | 1.05E-06 |
| GO:0099094 | Ligand-gated cation channel activity | 3 of 89 | 1.53 | 0.0093 |
| GO:0022836 | Gated channel activity | 8 of 313 | 1.41 | 3.42E-07 |
